# Arcyriaflavin A, a cyclin D1/CDK4 inhibitor, suppresses tumor growth, migration, and invasion of metastatic melanoma cells

**DOI:** 10.1186/s12935-025-03675-4

**Published:** 2025-02-13

**Authors:** Dokyeong Kim, Junseong Park, Yoon-Seob Kim, Okcho Na, Minyoung Park, Songzi Zhang, Sumin Cho, Yeun-Jun Chung

**Affiliations:** 1 Precision Medicine Research Center, College of Medicine, The Catholic University of Korea, Seoul, Republic of Korea; 2 Department of Medical Sciences, Graduate School of The Catholic University of Korea, Seoul, Republic of Korea; 3 Cancer Evolution Research Center, College of Medicine, The Catholic University of Korea, Seoul, Republic of Korea; 4 Department of Dermatology, Bucheon St. Mary’s Hospital, College of Medicine, The Catholic University of Korea, Seoul, Republic of Korea; 5 Department of Microbiology, College of Medicine, The Catholic University of Korea, 222 Banpo-daero, Seocho-gu, Seoul, 06591 Republic of Korea

**Keywords:** Anti-cancer therapy, Arcyriaflavin A, Cyclin D1, CDK4, Metastatic melanoma

## Abstract

**Background:**

Despite advancements in targeted therapy and immunotherapy, cutaneous melanoma continues to have a high mortality rate and poor prognosis, with therapies having limited efficacy in advanced melanoma. Therefore, it is crucial to develop novel therapeutics with proven clinical potential. In this study, we evaluated the efficacy of arcyriaflavin A (ArcA), a potent inhibitor of the cyclin D1/CDK4 complex, in suppressing aggressive phenotypes of metastatic melanoma.

**Methods:**

The effects of ArcA on viability and cell cycle were evaluated across four melanoma cell lines: WM239A and its metastatic derivatives: 113–6/4L, 131/4-5B1, and 131/4-5B2. Additionally, we performed wound healing and transwell invasion assays, followed by western blot. We further established xenograft mouse models by subcutaneously injecting them with the four melanoma cell lines and measured tumor size and weight biweekly. Immunohistochemistry analysis was performed to compare protein expression.

**Results:**

ArcA demonstrated dose-dependent cytotoxicity, selectively targeting melanoma cells without affecting normal cells, and induced G_1_ cell cycle arrest. Moreover, ArcA significantly inhibited cell migration and invasion in metastatic melanoma cell lines, accompanied by reduced expression levels of p-GSK-3β (Ser9), MMP-9, and MMP-13, suggesting that its anti-metastatic effects may be partially mediated through GSK-3β, MMP-9, and MMP-13. These findings were further validated using mouse xenograft models; ArcA-treated mice exhibited significantly smaller tumor volumes and lighter tumor weights compared to vehicle-treated mice. Immunohistochemistry further confirmed decreased expression of p-GSK-3β, MMP-9, and MMP-13 in tumor tissues from ArcA-treated mice.

**Conclusions:**

Collectively, our findings indicate that ArcA possesses substantial anti-tumor potential, including cytotoxic effects and inhibition of migration and invasion in metastatic melanoma. These results suggest that ArcA could enhance therapeutic efficacy in the treatment of metastatic melanoma.

**Supplementary Information:**

The online version contains supplementary material available at 10.1186/s12935-025-03675-4.

## Background

Although malignant melanoma comprises approximately 5% of all skin cancers, it accounts for > 75% of skin cancer-related deaths, particularly among patients with metastatic melanoma [1]. Recent targeted therapies and immunotherapies have significantly transformed the treatment landscape for advanced melanoma [2, 3]; however, over 40% of patients still fail to respond to these therapies [4, 5]. This underscores the urgent, unmet need to identify and develop novel therapeutic approaches for patients with advanced melanoma.

Cyclin D1 plays a pivotal role in promoting cell cycle progression by facilitating the transition from the G_1_ to the S phase at the restriction point, working in conjunction with the allosteric regulator of CDK4 and CDK6 [6]. The overexpression of cyclin D1 has been widely reported in various cancers, including breast, lung, colon, and head and neck cancers [7]. Notably, abnormalities in the cyclin D1-CDK4/6 complex have been implicated in 35–90% of melanomas, and their oncogenic alterations are associated with decreased survival and therapeutic resistance [8, 9]. Correspondingly, our previous studies utilizing single-cell RNA sequencing revealed a progressive upregulation of cyclin D1 in metastatic melanoma cells that had spread to the lung and brain compared to their matched primary melanoma cells [10]. These findings collectively highlight the critical role of cyclin D1 in melanoma progression.

Arcyriaflavin A (ArcA), an indolocarbazole alkaloid derived from the mycomycetes *Arcyria obvelata* and *Arcyria denudate*, is a well-known inhibitor of the cyclin D1-CDK4 complex [11]. Research has demonstrated its ability to inhibit the replication of human cytomegalovirus (HCMV) [12] and its anti-cancer activity in various solid cancers [13–15]. Despite these findings, its anti-cancer effects and the underlying molecular mechanisms in melanoma remain unexplored.

In this study, we aimed to evaluate the anti-cancer efficacy of ArcA as a cyclin D1/CDK4 inhibitor in metastatic melanoma cells, specifically using highly metastatic derivatives that had metastasized to the lung and brain [16]. Our findings demonstrated that ArcA significantly inhibited cell proliferation, migration, and invasion in these metastatic melanoma cells, both in vitro and in vivo. These results highlight the anti-tumor potential of ArcA, suggesting that it could enhance therapeutic efficacy for patients with advanced melanoma.

## Methods

### Cell culture

The WM239A human melanoma cell line and its highly metastatic variants, namely the 113/6–4 L lung-metastatic cell line and brain-metastatic cell lines 131/4-5B1 and 131/4-5B2 [16], were cultured in RPMI-1640 medium (HyClone, Logan, UT, USA). Based on the previously analyzed scRNA-seq data [10], we verified cross-contamination and tissue specificity of the four melanoma cell lines using the TiGER database (Fig. S1) [17]. The HEK293 normal human kidney cell line was cultured in EMEM medium (Quality Biological, Gaithersburg, MD, USA). HEL299 normal human lung fibroblasts, Detroit 551 normal human skin fibroblasts, and B16-F10 mouse melanoma cell line were cultured in RPMI-1640 medium. All media contained 10% fetal bovine serum (FBS; HyClone) and 1% penicillin-streptomycin (Sigma-Aldrich, Darmstadt, Germany). Cells were maintained in a controlled environment at 37 ℃ with 5% CO_2_, and the medium was changed every 2 to 3 days. All experiments were conducted using mycoplasma-free cell lines, verified routinely for contamination using a mycoplasma PCR detection kit (Applied Biological Materials Inc., Richmond, BC, Canada).

### Cell cytotoxicity assay

Cell viability in response to ArcA was assessed using WST assays with the Cell Counting Kit-8 (CCK-8; Dojindo Molecular Technologies, Kumamoto, Japan). Briefly, cells were seeded in 96-well plates (SPL, Pocheon, Republic of Korea) at a density of 8 × 10^3^ cells per well and treated with varying concentrations of ArcA for 72 h. After incubation, CCK-8 solution (10% of the medium volume) was added, and cells were incubated for an additional 1.5 h. Absorbance was measured at 450 nm using a microplate reader (BioTek Instruments Inc., Santa Clara, CA, USA). The half-maximal inhibitory concentration (IC_50_) was calculated using nonlinear regression analysis of the WST results with GraphPad Prism 8.0. (GraphPad Software, Boston, MA, USA).

### Cell cycle analysis

Cell cycle analysis was performed using propidium iodide (PI) staining. Cells were plated at a density of 1 × 10^5^ cells per well in 6-well plates (SPL) and allowed to stabilize overnight. The following day, cells were treated with ArcA (10 µM) for 18 h. After treatment, cells were harvested, fixed in 70% ethanol, and centrifuged. The cell pellets were then incubated with RNase A solution (100 µg/mL, Roche, Basel, Switzerland) for 15 min, followed by staining with PI solution (50 µg/mL). Cell cycle distribution was analyzed using flow cytometry (FACS Canto, BD, USA).

### Wound healing assay

For the scratch wound-closure assay, cells were seeded into 6-well plates (SPL) and cultured until reaching approximately 90% cell confluence. A perpendicular scratch was made on the cell monolayer using 200 µL pipette tips. Detached cells were removed by gently washing with phosphate-buffered saline (PBS; HyClone), and the remaining cells were incubated in serum-free medium. Bright-field images were obtained at 0 and 48 h, and wound closure was quantified using ImageJ software.

### Transwell invasion and migration assay

Transwell invasion and migration assays were performed using 8-µm pore Millicell inserts (SPL). For the invasion assay, inserts were pre-coated with Matrigel (Corning, New York, NY, USA) diluted in serum-free RPMI medium at a 1:4 ratio and placed into 24-well plates (SPL). For transwell migration assay, inserts without Matrigel coating were used. In the upper chamber, 100 µL of serum-free medium containing 2 × 10^4^ cells was added, while the lower chamber was filled with 500 µL of medium containing 10% FBS. The plates were incubated for 24 h to allow cells to attach to the bottom of the chambers, following which ArcA and control diluent (DMSO) were added to the upper chamber. Following a 48 h incubation, non-invading or non-migrating cells remaining in the upper chamber were carefully removed. Cells that migrated or invaded into the lower chamber were defined as invaded/migrated cells. These cells were fixed with 4% had paraformaldehyde and stained with 0.5% crystal violet (Sigma-Aldrich). Membrane images were captured using a Pannoramic SCAN II (3DHISTECH Ltd., Budapest, Hungary), and cells were quantified across at least three randomly selected fields per slide at 20× magnification.

### Protein extraction and western blot

Total proteins were extracted using ice-cold RIPA lysis buffer (Biosesang, Seongnam, Republic of Korea), containing a protease inhibitor (GenDEPOT, Katy, TX) and phosphatase inhibitor cocktail (GenDEPOT). Protein concentrations were quantified using bovine serum albumin as the standard (BIO-RAD, Hercules, CA, USA). Protein samples (20 µg) were subjected to SDS-PAGE and transferred to PVDF membranes (Millipore, Billerica, MA, USA). To reduce background staining, membranes were blocked with 5% non-fat dry milk for 1.5 h. Membranes were then immunoblotted overnight at 4 °C with the following antibodies: anti-β-Actin (1:1000, Cell Signaling Technology, Boston, MA, USA), anti-phospho-GSK-3β (p-GSK-3β, ser9; 1:1000, Cell Signaling Technology), anti-t-GSK-3β (1:1000, Cell Signaling Technology), anti-phospho-Rb (ser807/811, 1:1000, Cell Signaling Technology), anti-t-Rb (1:2000, BD Biosciences), anti-CyclinD1 (1:1000, Cell Signaling Technology), MMP9 (1:1000, Cell Signaling Technology), and MMP13 (1:2000, ABclonal, Woburn, MA). After washing with Tris-buffered saline with Tween-20 (TBST), membranes were incubated with anti-rabbit IgG-HRP (1:4000, Santa Cruz Biotechnology, Dallas, TX, USA) for 1 h at room temperature. Chemiluminescence signals were detected using Pico PLUS or Femto Maximum Sensitivity Substrate (Thermo Scientific, Waltham, MA, USA) and quantified using the Amersham Imager 600 (Fujifilm, Tokyo, Japan). The results were presented as fold changes by normalizing the target expression levels to those of β-Actin for each sample.

### RNA extraction and quantitative real-time PCR (qRT-PCR)

cDNA was synthesized from total RNA using the SuperScript™ VI First-Strand Synthesis System (Invitrogen, MA, USA) according to the manufacturer’s instructions. Real-time PCR was performed using the BioRad CFX96 system (BioRad, CA, USA) using THUNDERBIRD™ SYBR^®^ qPCR Mix (TOYOBO, Osaka, Japan). Primer sequences used are as follows: MMP-9: forward (F): 5′-TTCCAAACCTTTGAGGGCGA-3′, reverse (R): 5′-CAAAGGCGTCGTCAATCACC-3′; MMP-13: F: 5′-AACGCCAGACAAATGTGACC-3′, R: 5′-AGGTCATGAGAAGGGTGCTC-3′. Human glyceraldehyde 3-phosphate dehydrogenase (hGAPDH) served as the housekeeping gene. The thermal cycling conditions were as follows: an initial denaturation step at 95 °C for 1 min, followed by 40 cycles at 95 °C for 5 s, 54–58 °C for 10 s, and 72 °C for 20 s. Each data point was analyzed in triplicate. Relative gene expression was quantified using the 2^−ΔΔCt^ method.

### Mouse xenograft model

Five-week-old healthy male BALB/c nude mice, weighing 18–20 g, were obtained from Orient Bio Laboratories (Seoul, Republic of Korea). The mice were housed under standard laboratory conditions with a 12 h light-dark cycle and maintained at an appropriate temperature, humidity, and ventilation. After a one-week acclimatization period, the mice were randomly assigned to four groups for the in vivo tumor growth study: vehicle or ArcA. Each mouse received a subcutaneous injection of 5 × 10^5^ cells (mixed with Matrigel/serum-free RPMI at a 1:2 ratio) into the right flank. Once the tumors became palpable, mice were treated intratumorally with 75 µg/100 mm^3^ of ArcA, while the vehicle group received an equivalent volume of DMSO. Injections were administered twice a week. Tumor sizes were measured using calipers, and tumor volume (V) was calculated using the formula V = 1/2 × length × width × width (mm^3^). Both body and tumor weight were recorded. After two weeks, the mice were euthanized by CO_2_ inhalation to minimize discomfort. Tumors were then dissected and collected for further analysis. All animal procedures followed institutional guidelines and were approved by the Institutional Animal Cancer and Use Committee of the Catholic University of Korea (CUMS-2021-0352-02).

### Tissue processing and immunohistochemistry (IHC)

Fresh tumor tissues were fixed in 4% paraformaldehyde for at least 24 h, then embedded in paraffin, sectioned, and prepared for staining. Tissue Sect. (4 μm) were deparaffinized with Neo-Clear xylene substitute (Sigma-Aldrich) and rehydrated through a graded series of ethanol dilutions (Sigma-Aldrich).

For IHC, the following primary antibodies were used: p-GSK-3β (1:200, Cell Signaling Technology), MMP-9 (1:500, ABclonal), and MMP-13 (1:500, ABclonal). After incubation with the primary antibodies, samples were treated with the corresponding secondary antibody (Dako Agilent, Santa Clara, CA). Following washes with TBST (Biosesang), specimens were incubated in DAB solution for 1–10 min and then stained with hematoxylin for a further 1 min. Images were captured using a Pannoramic SCAN II, with at least three randomly selected fields captured per tissue section.

### Statistical analysis and software

Statistical analyses were performed using GraphPad Prism, including Student’s *t*-test, one-way ANOVA, and repeated measures ANOVA, to determine statistical significance. Results are presented as the mean ± standard error of the mean (SEM).

## Results

### ArcA exhibits cytotoxic effects in metastatic melanoma cell lines

**Fig. 1:**
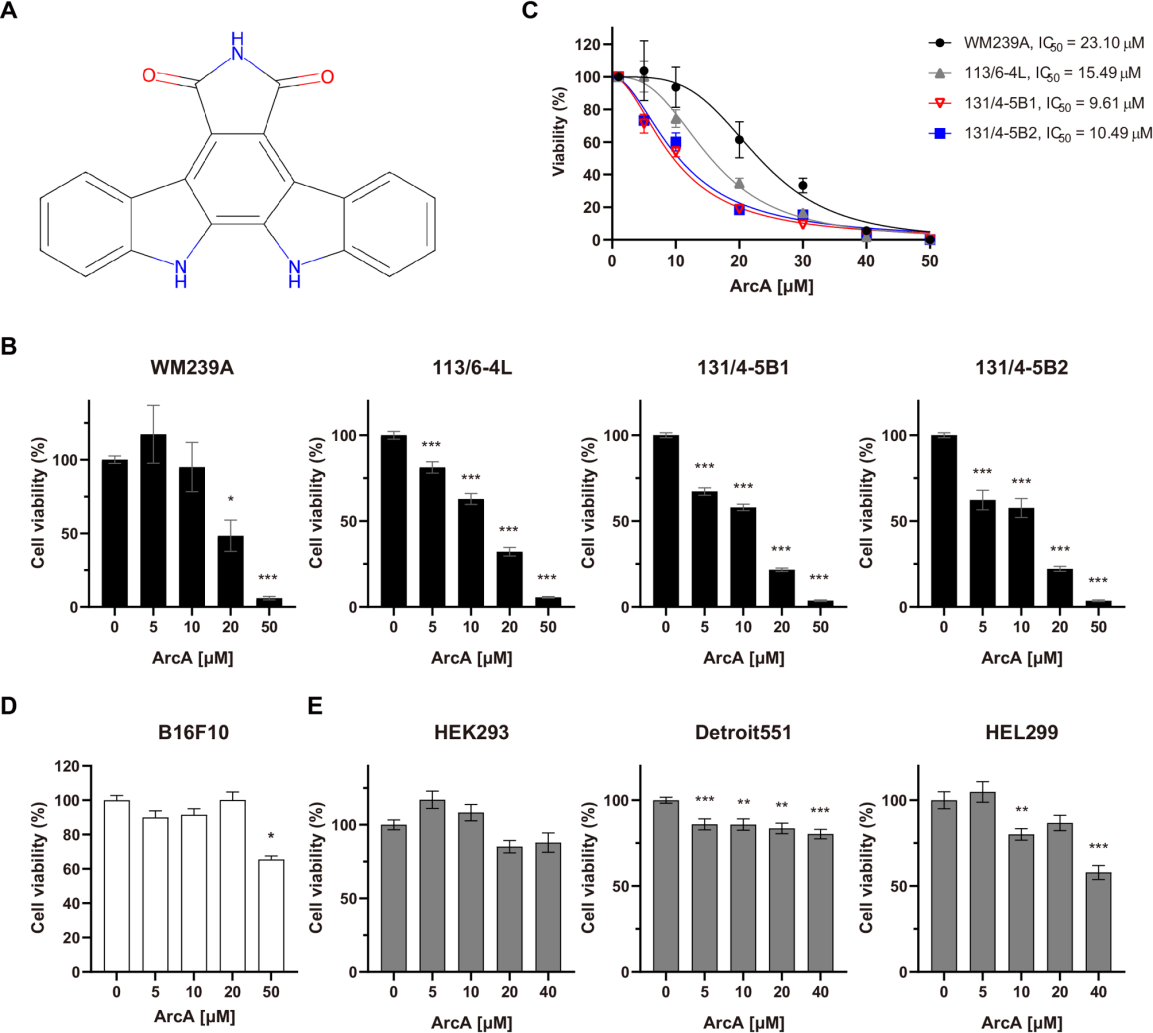
Cytotoxic effects of arcyriaflavin A (ArcA) on metastatic melanoma cell lines. (**A**) The chemical structure of ArcA, as retrieved from the ChemSpider chemical database. (**B**, **C**) Viability of the metastatic cell lines WM239A, 113/6–4 L, 131/4-5B1, and 131/4-5B2 after 72 h of treatment with various concentrations of ArcA, assessed using WST assays (*n* = 7 per cell line). The results are displayed as bar graphs (**B**) and dose-response curves (**C**). Viability of B16F10 cell lines, which express wild-type *BRAF*, treated with various concentrations of ArcA (*n* = 6). (**E**) Viability of normal cell lines, including HEK293 human embryonic kidney cells, Detroit551 human skin fibroblasts, and HEL299 human lung fibroblasts, treated with various concentrations of ArcA (*n* = 6 per cell line). DMSO was used as the control diluent in all viability experiments, as it as the solvent used to dissolve ArcA. Statistical analysis was performed using one-way ANOVA, followed by Dunnett’s multiple comparisons test. Data are expressed as the mean ± SEM, with statistical significance indicated by asterisks (*P < 0.05, **P < 0.01, *** *P* < 0.001). Asterisks above treatment groups indicate the p-value compared to controls

We initially evaluated the effects of ArcA (Fig. 1A) on cell viability across four melanoma cell lines: WM239A and its metastatic derivatives: 113–6/4L, 131/4-5B1, and 131/4-5B2. After 72 h of treatment, ArcA induced a dose-dependent decrease in viability across all melanoma cell lines (Fig. 1B). Notably, the metastatic sublines 113/6–4 L, 131/4-5B1, and 131/4-5B2 exhibited greater sensitivity to ArcA compared to the primary cell line WM239A (Fig. 1C). To investigate whether ArcA’s cytotoxic effects were associated with *BRAF* mutations, we assessed its impact on B16F10 cells, which express wild-type *BRAF* [18]. Unlike WM239A and its variants, B16F10 cells exhibited more resistance to ArcA, with significant reduction in viability observed only at concentrations exceeding 50 µM (Fig. 1D). These findings suggest that ArcA’s cytotoxic effects are more pronounced in melanoma cell lines harboring *BRAF* mutations. We further evaluated the effects of ArcA on normal cell lines, including the HEK293 human embryonic kidney cell line, Detroit551 skin fibroblasts, and HEL299 lung fibroblasts. These non-cancerous cell lines did not exhibit noticeable growth suppression regardless of treatment dose (Fig. 1E), maintaining over 80% cell viability at concentrations below 20 µM (Fig. 1E). Collectively, these results indicate that ArcA exhibits potent cancer-specific cytotoxic effects, with enhanced efficacy in *BRAF*-mutated melanoma cells. To ensure sublethal exposure for subsequent functional assays, we selected a treatment concentration of 10 µM for 48 h, which is below the IC_50_ values for all melanoma cell lines (Fig. 1B, C).

### ArcA induces G1 cell cycle arrest in metastatic melanoma cell lines

**Fig. 2:**
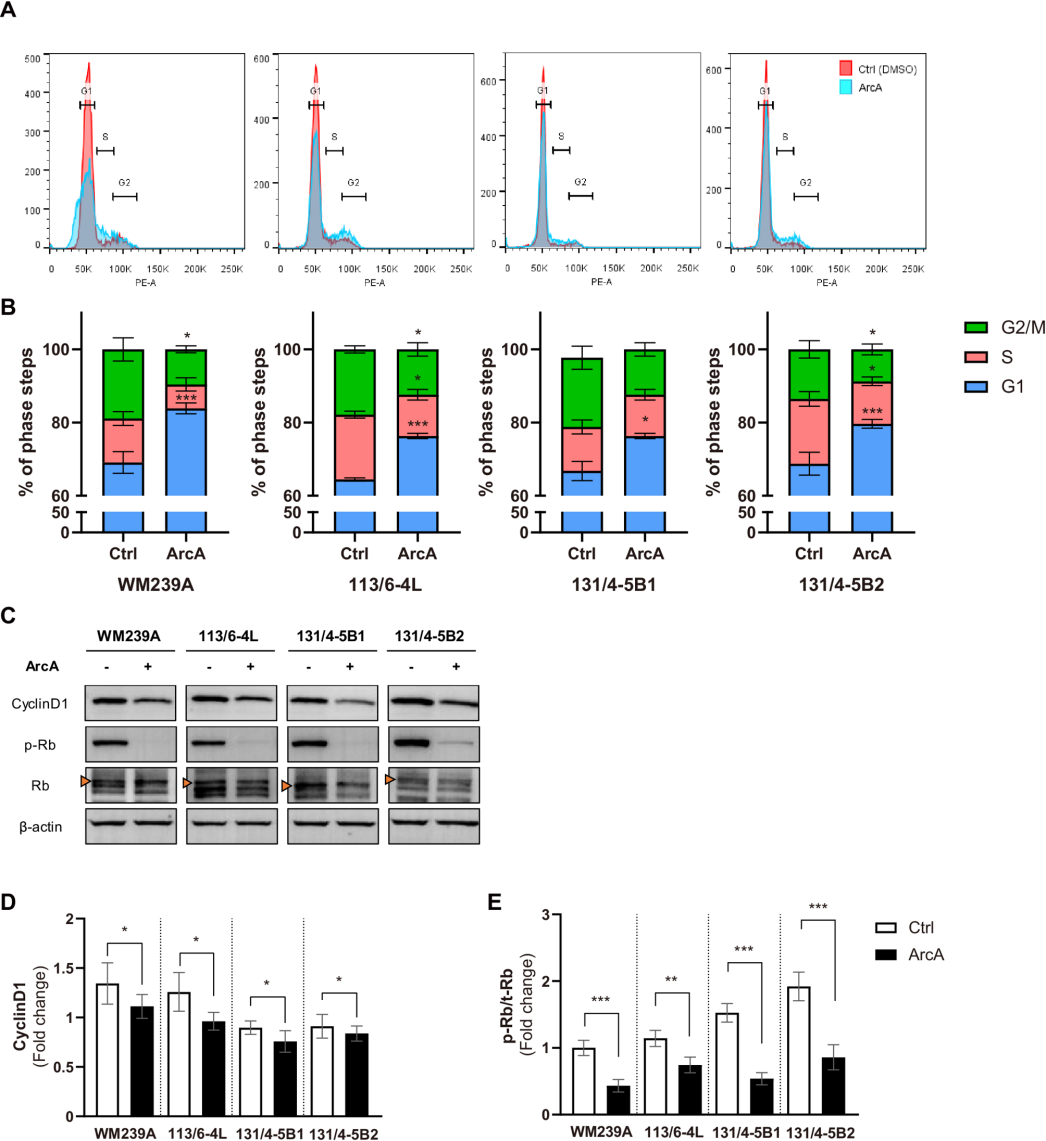
The effect of ArcA on G_1_ cell cycle arrest in metastatic melanoma cell lines. (**A**) Flow cytometry analysis of propidium iodide-stained cells to evaluate cell cycle distribution following an 18 h treatment with 10 µM ArcA or an equivalent volume of DMSO (control). Upper panels show histograms of cell cycle phases, and lower panels present the percentage of cells in each phase as bar graphs. Analysis was performed in triplicate, with data expressed as the mean ± SEM. Statistical significance was assessed using two-way ANOVA, followed by Bonferroni’s multiple comparisons test, with comparisons made to the control group (*P < 0.05, *** *P* < 0.001). (**B**) Western blot analysis of cyclin D1, phosphorylated Rb (p-Rb, ser807/811), and total-Rb in melanoma cells treated with 10 µM ArcA or DMSO (control). Orange arrows indicate the specific bands among multiple bands. (**C**,** D**) Quantification of the western blot results, showing the expression levels of cyclin D1 (**C**) and p-Rb (**D**) normalized to β-actin, with p-Rb/actin further normalized to t-Rb/actin. Data represent the mean ± SEM from three independent experiments, with statistical significance determined using a *t-*test (*P < 0.05, **P < 0.01, and ***P < 0.001). All experiments were performed in triplicate to ensure reproducibility

We next examined the growth-inhibitory effects of ArcA in metastatic melanoma cell lines using cell cycle analysis, which enabled us to quantify the distribution of cells across different phases of the cell cycle. For this, the four melanoma cell lines were treated with ArcA for 18 h and stained with PI. ArcA treatment significantly increased the percentage of cells in the G_1_ phase to 83.9% in WM239A, 76.3% in 113/6–4 L, 75.7% in 131/4-5B1, and 81.6% in 131/4-5B2, compared to the control groups (Fig. 2A). To explore the molecular mechanisms underlying G_1_ phase accumulation, we analyzed the expression levels of key regulators involved in cell cycle progression using western blotting. ArcA treatment markedly reduced the protein levels of cyclin D1, a critical driver of the G_1_/S transition (Fig. 2B, C). In addition, the level of phosphorylated retinoblastoma protein (p-Rb), a downstream target of cyclin D1/CDK4/6 pathway, was significantly decreased in all four cell lines following ArcA treatment (Fig. 2B, D). These results indicate that ArcA effectively induces G_1_/S phase cell cycle arrest by suppressing cyclin D1 expression and reducing Rb phosphorylation, thereby inhibiting the proliferation of melanoma cells.

### ArcA inhibits migratory and invasive capacities in metastatic melanoma with decreased the expression of p-GSK-3β/MMP-9/MMP-13

**Fig. 3:**
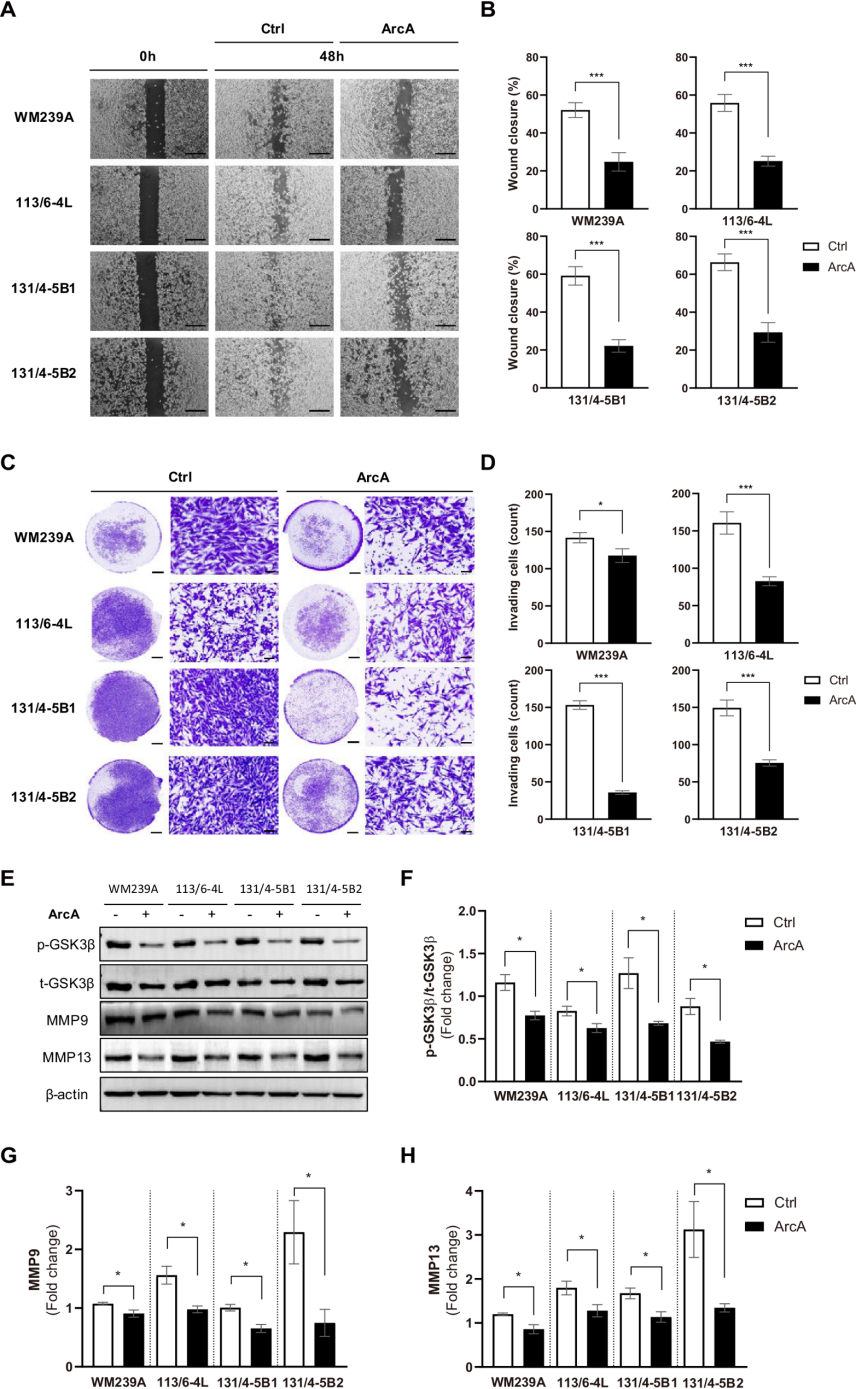
Inhibition of migratory and invasive properties by ArcA with decreased p-GSK-3β Ser9, MMP-9, and MMP-13. (**A**) Wound healing assay illustrating the effect of ArcA on cell migration at 48 h post-scratch. Scale bars: 500 μm. (**B**) The bar graph shows the percentage of wound closure relative to the control. Data are presented as the mean ± SEM from three independent experiments. Statistical significance was assessed using a *t-*test (*P < 0.05, *** *P* < 0.001). (**C**) Transwell invasion assay indicating a significant reduction in the number of invaded cells following ArcA treatment. Lower magnification (1.5×) images are shown in the left panels, and higher magnification (20×) images are shown in the right panels. Scale bars: 1000 μm for lower magnification (left panels) and 50 μm for higher magnification (right panels). (**D**) Bar graphs showing the number of invaded cells in at least three randomly selected fields (20× magnification). Data are expressed as the mean ± SEM from three independent experiments. Statistical significance was determined using a *t-*test (*P < 0.05, ***P < 0.001). (**E**) Western blot analysis of the expression levels of p-GSK-3β Ser9 (p-GSK-3β; Ser9), total-GSK-3β (t-GSK-3β), MMP9, MMP13, and β-actin in ArcA-treated melanoma cells. (**F-H**) Quantification of protein expression levels from western blots: (**F**) p-GSK-3β relative to t-GSK-3β, normalized to β-actin. (**G**) MMP9 and (**H**) MMP13, normalized to β-actin. Data represents the mean ± SEM of three independent experiments. Statistical significance was determined by a *t-*test (*P < 0.05)

To determine whether ArcA exerts anti-metastatic activity on metastatic melanoma cells, we performed wound healing and transwell invasion assays at 48 h post-treatment when cytotoxic effects are negligible (Fig. S2). Control cells (DMSO-treated cells) in the cell monolayer exhibited faster migration, effectively closing the scratch gap, whereas ArcA-treated cells exhibited a significantly larger wound area (Fig. 3A, B). Similarly, the results of transwell migration assay were consistent with those of the wound healing assay (Fig. S3). Additionally, transwell invasion assays demonstrated that ArcA treatment markedly reduced the number of invaded cells across all four melanoma cell lines (Fig. 3C, D).

To further investigate the molecular mechanism underlying the effects of ArcA in metastatic melanoma, we examined its impact on glycogen synthase kinase-3β (GSK-3β), a protein crucial for cell proliferation and metastasis [19]. Western blot analysis revealed that ArcA treatment led to a marked reduction in the levels of phosphorylated GSK-3β (p-GSK-3β) at Ser9 across all four melanoma cell lines (Fig. 3E, F). Protein quantification further confirmed this, revealing a significant decrease in the ratio of p-GSK-3β to total GSK-3β following treatment. Moreover, we assessed the expression of matrix metalloproteinases (MMPs), specifically MMP-9 and MMP-13. As anticipated, ArcA treatment resulted in decreased protein and mRNA levels of both MMP-9 and MMP-13 across all melanoma cell lines (Fig. 3E and G-H and Fig. S4). These findings suggest that the anti-metastatic effects of ArcA in melanoma cells are attributed to the dephosphorylation of GSK-3β at Ser9, along with the subsequent reduction in MMP-9 and MMP-13 levels.

### ArcA suppresses tumor growth in xenograft models of melanoma

**Fig. 4:**
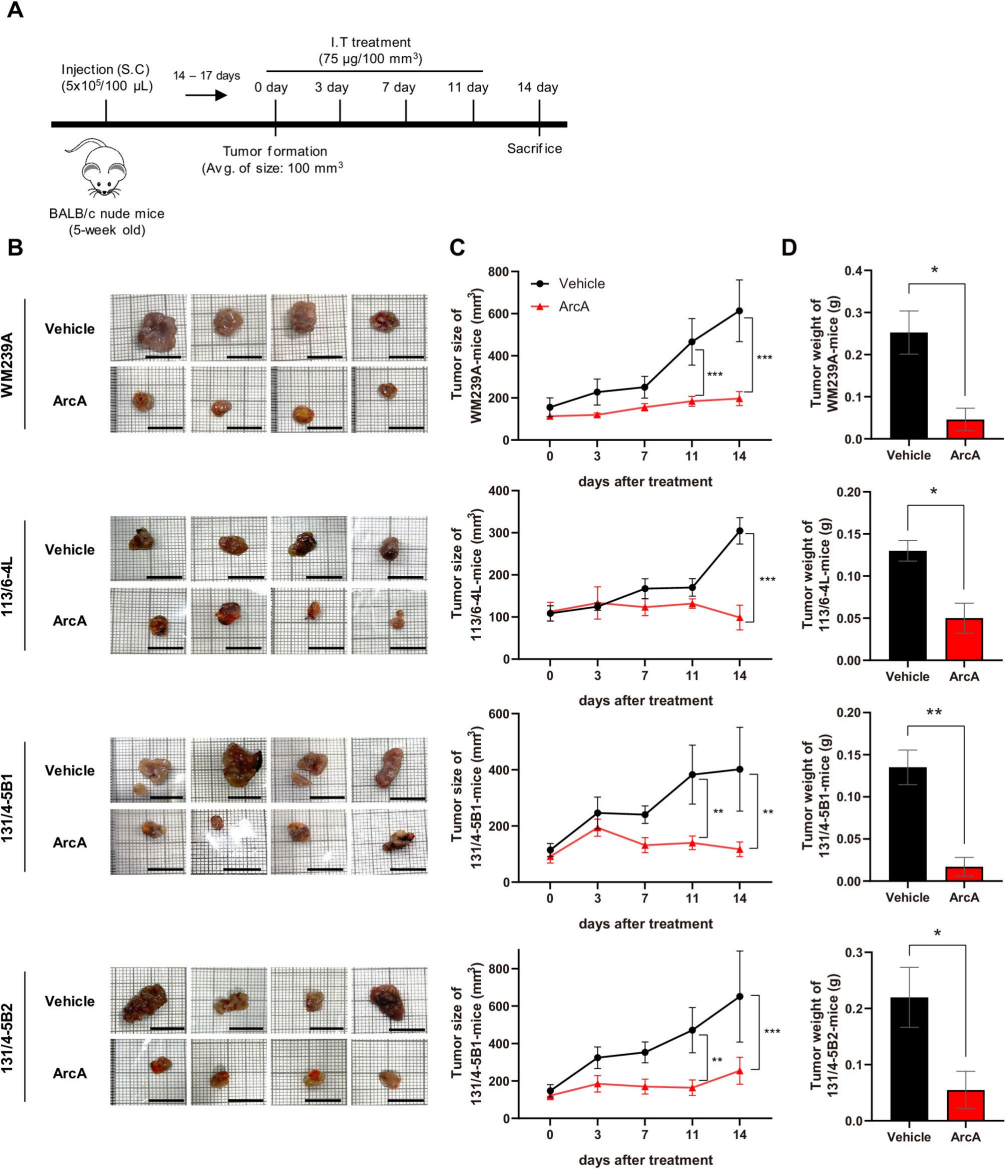
In vivo anti-cancer efficacy of ArcA in xenograft mouse models. (**A**) A schematic representation of the experimental timeline detailing the establishment of the xenograft models and the ArcA treatment protocol. (**B**) Representative images of tumors excised from each mouse (scale bar: 1 mm). (**C**) Tumor growth curves showing the average tumor size in each group (*n* = 4 per group). Statistical significance was determined using repeated measures ANOVA followed by Bonferroni’s multiple comparisons test (**P < 0.01; ***P < 0.001). (**D**) Tumor weights post-sacrifice, shown as bar graphs. Statistical significance was evaluated using a *t-*test (*P < 0.05, ** *P* < 0.01)

To evaluate the efficacy of ArcA in vivo, we established xenograft mouse models by subcutaneously injecting them with four melanoma cell lines. Once the average tumor size reached 100 mm^3^ (14–17 days post-inoculation), the mice were treated intratumorally with ArcA twice a week for 2 weeks (Fig. 4A). Remarkably, ArcA treatment resulted in a significant reduction in tumor size compared to that in vehicle-treated groups (Fig. 4B, C), with no observable systemic toxicity (Fig. S5). Consistent with these findings, the tumor weights of ArcA-treated mice were lower than those in the vehicle-treated mice (Fig. 4D).

**Fig. 5:**
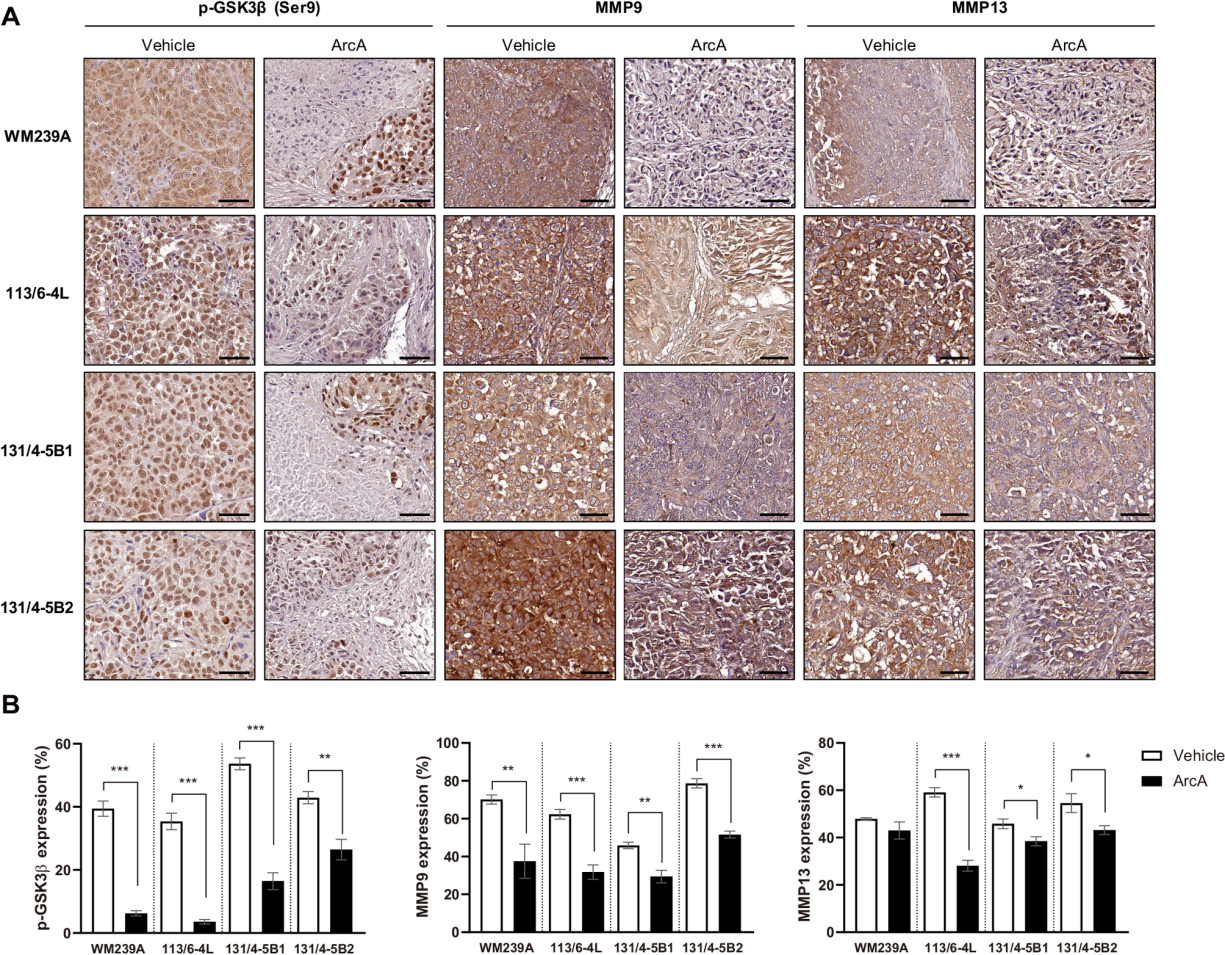
Downregulation of p-GSK-3β Ser9, MMP-9, and MMP-13 expression by ArcA in mouse xenograft models. (**A**) Immunohistochemical (IHC) analysis showing the expression levels of p-GSK-3β, MMP-9, and MMP-13 in tumor tissues from vehicle- and ArcA-treated mice (*n* = 4 per group). Representative images are shown at 20× magnification (scale bar: 50 μm). (**B**) Quantified bar graphs corresponding to the IHC images, based on at least three randomly selected fields (20× magnification). The bar graphs illustrate the percentage of positive staining per area. Data are presented as the mean ± SEM. Statistical analysis was performed using a *t*-test, with significance levels indicated as *P < 0.05, **P < 0.01, and ***P < 0.001

IHC analysis was performed to compare the expression levels of GSK-3β, MMP-9, and MMP-13 between vehicle- and ArcA-treated mice. As expected, the expression levels of p-GSK-3β (Ser9), MMP-9, and MMP-13 were notably reduced in the tissues of ArcA-treated mice (Fig. 5A). The quantified bar graphs corroborated the IHC images (Fig. 5B). These findings demonstrate that ArcA treatment effectively inhibits melanoma tumor growth in vivo, and this is associated with the downregulation of p-GSK-3β, MMP-9, and MMP-13.

## Discussion

Our previous studies performed single-cell RNA sequencing on the WM239A melanoma cell line and its highly metastatic sublines, which spread to the lung (113/6–4 L) and brain (131/4-5B1 and 131/4-5B2) [10]. These sublines were established several months after the subdermal injection of WM239A cells into severe combined immunodeficient mice [16]. This paired model provides a valuable framework for understanding the molecular-genetic changes during the transition from primary to distant metastatic phenotypes; accordingly, our previous findings identified that cyclin D1 is a key factor that drives the distant metastasis of melanoma [10]. Cyclin D1 promotes uncontrolled cell proliferation, a hallmark of cancer, and its amplification has been reported in up to 90% of melanoma cases [6, 8, 20]. An elevated CCND1 copy number is defining characteristic of malignant melanoma and correlates with poor prognosis in melanoma patients [21, 22]. Herein, this study investigated the efficacy of ArcA, a potent inhibitor of the cyclin D1/CDK4 complex, in metastatic melanoma cells. Our findings demonstrate significant anti-tumor potential, including inhibited cell proliferation, migration, and invasion.

Cyclin D1/CDK inhibitors have been evaluated across various phases of clinical trials for both cancers and other diseases [23]. Notably, various third-generation, FDA-approved cyclin D1/CDK inhibitors such as palbociclib, ribociclib, and abemaciclib are currently undergoing preclinical studies in patients with melanoma [24, 25]. Multiple preclinical trials have demonstrated the anti-tumor potential of these inhibitors, particularly when combined with immunotherapy and targeted therapy. For instance, palbociclib has been demonstrated to enhance the efficacy of anti-PD-1 immunotherapy [26], while abemaciclib can suppress immune-resistant pathways, thereby sensitizing tumors to anti-CTLA-4 and anti-PD-1 therapies [27]. Palbociclib has been demonstrated to enhance the therapeutic efficacy of vermurafenib and trametinib in *BRAF*-mutant melanoma cells [28–30]. In addition to melanoma, ribociclib with encorafenib have synergistic cytotoxic effects in *BRAF*-mutant colorectal cancer [31]. However, despite these observed synergistic effects, cyclin D1/CDK inhibitors frequently induce drug resistance pathways, such as MAP kinase and PI3K-AKT-mTOR signaling, when used alone [32]. In contrast, our findings indicate that ArcA exhibits cancer-specific cytotoxicity and anti-metastatic activity in vitro, with GSK-3β dephosphorylation at Ser9, a process negatively regulated by MAP kinase and PI3K-AKT-mTOR signaling [19]. Furthermore, ArcA reduces tumor growth in vivo without causing systemic toxicity in the short-term. These results suggest that ArcA serves as a promising cyclin D1/CDKs inhibitor with potential to overcome resistance as a single agent or in combination therapy for metastatic melanoma. However, several important questions remain regarding the efficacy and molecular mechanisms of ArcA. Our study primarily focused on melanoma cell lines with specific genetic backgrounds, particularly those harboring a *BRAF* V600D mutation. Melanoma is a highly heterogeneous disease, and its genetic and molecular diversity may affect the therapeutic response to ArcA. Although we demonstrated the less cytotoxic effects to ArcA on melanoma with wild-type *BRAF* using B16F10 cell lines, there are limitations related to species differences, which complicate the interpretation of sensitivity variations by ArcA based on *BRAF* mutation status. Therefore, it is crucial to evaluate the effectiveness of ArcA in a broader panel of melanoma models, including human and patient-derived melanoma cell lines with various genetic profiles. Moreover, to date, research on the anti-cancer effects of ArcA primarily focusing on a few cancer cell lines in laboratory settings, and there is only one in vivo study using a chick embryo model [13–15]. Hence, further investigation is crucial to fully assess its potential for clinical use.

GSK-3β, a ubiquitously expressed serine/threonine kinase crucial for regulating glycogen synthesis, has emerged as a promising target for cancer treatment, including melanoma, owing to its role in key pathways modulating cell survival, proliferation, and drug resistance [19]. A key finding of this study is the ArcA-mediated reduction in GSK-3β phosphorylation at Ser9. The activity of GSK-3β is intricately regulated by its phosphorylation status: phosphorylation at Ser9 inhibits GSK-3β activity, while phosphorylation at Tyr216 enhances it [33]. Therefore, ArcA promotes GSK-3β activation through Ser9 dephosphorylation. Interestingly, in melanoma, GSK-3β negatively regulates the stabilization of microphthalmia-associated transcription factor (MITF) as well as the Wnt/β-catenin and PI3K/AKT pathways, which promote cancer cell survival, proliferation, and resistance to apoptosis [19, 34, 35]. Additionally, GSK-3β activation facilitates the degradation of cyclin D1 [36]. Thus, the ArcA-induced dephosphorylation (Ser9) of GSK-3β may enhance the cell cycle inhibitory effects, thereby enhancing its cytotoxic effects against metastatic melanoma. However, GSK-3β has dual roles in various cancer types, acting as both a tumor suppressor and an oncogene [35, 37]. Therefore, we cannot exclude the potential for unintended pro-tumorigenic effects resulting from ArcA-mediated dephosphorylation of GSK-3β at Ser9 in different cancer types. Further investigation is needed to gain detailed insights into the mechanisms underlying ArcA-mediated GSK-3β activity and its tumor suppressive effects in metastatic melanoma. GSK-3β activity also plays a role in the negative regulation of MMPs, which are critical for the degradation of extracellular matrix and thus key in tumor invasion and metastasis [38–40]. Our results indicate that ArcA treatment leads to a reduction in expression levels of MMP-9 and MMP-13. The expression of both MMPs has been associated with melanoma progression [41, 42]. This suggests that the activation of GSK-3β by ArcA may suppress the transcription or activity of these MMPs, thereby inhibiting the invasive capabilities of melanoma cells.

## Conclusion

**Fig. 6:**
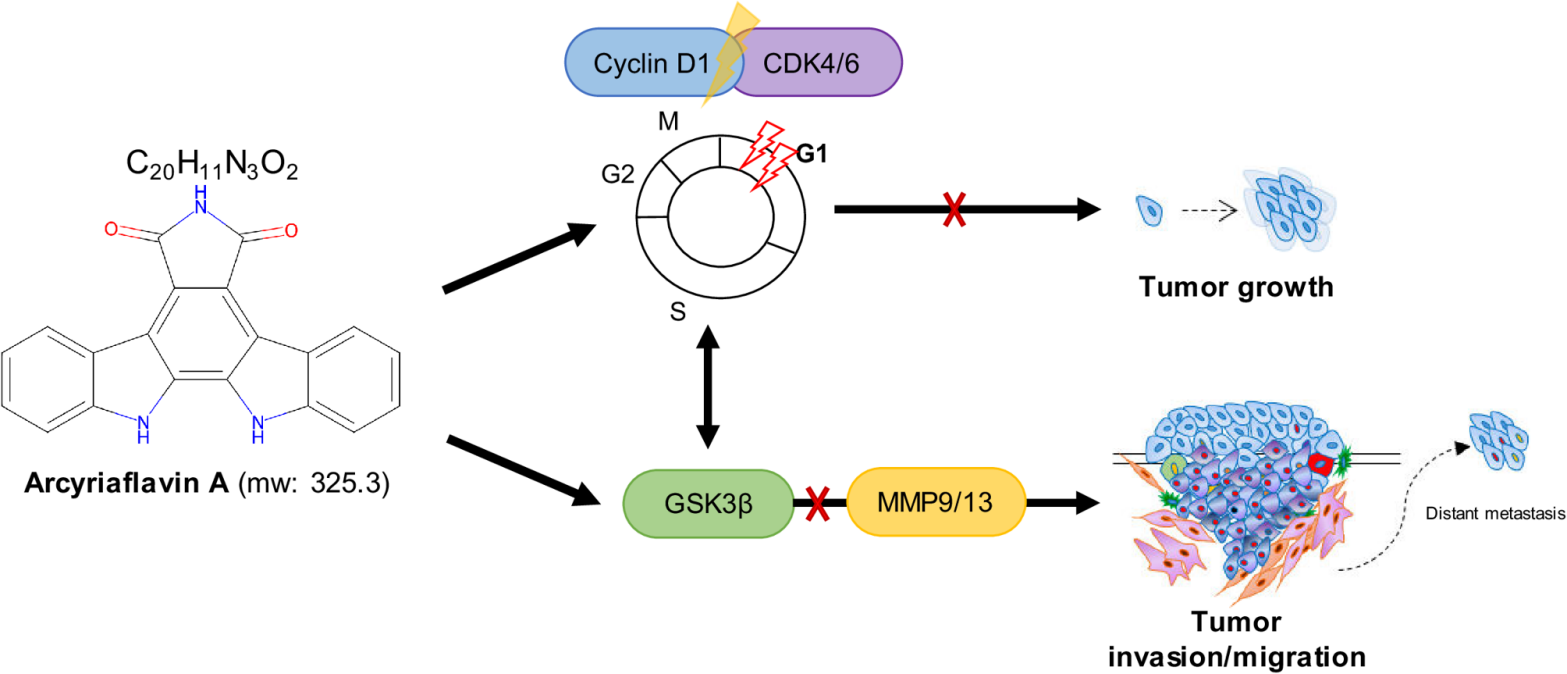
Schematic overview of the study. A proposed signaling pathway model illustrating the anti-tumor effects of ArcA on the aggressive behavior of metastatic melanoma cells

In conclusion, our study provides strong evidence that ArcA effectively suppresses key malignant behaviors, including proliferation, migration, and invasion, in metastatic melanoma cells (Fig. 6). Notably, this study is the first to demonstrate that ArcA, a potent cyclin D1/CDK4 inhibitor, dephosphorylates GSK-3β at Ser9 and reduces MMP-9 and MMP-13 expression, revealing a novel therapeutic mechanism. These findings position ArcA as a promising candidate for combination therapy, potentially minimizing redundancy in therapeutic approaches. Furthermore, we suggest that ArcA has substantial potential as a therapeutic agent for patients with advanced melanoma. Further studies are crucial to develop a clinically applicable therapeutic strategy that incorporates ArcA, with the potential to improve treatment outcomes in metastatic melanoma.

## Electronic supplementary material

Below is the link to the electronic supplementary material.

Supplementary Material 1

## Data Availability

No datasets were generated or analysed during the current study.

## Abbreviations

ArcA: Arcyriaflavin A
FBS: Fetal bovine serum
GSK-3β: Glycogen synthase kinase-3β
p-GSK-3β: Phosphorylated GSK-3β
PBS: Phosphate-buffered saline
TBST: Tris-buffered saline with Tween 20
Rb: Retinoblastoma protein
IHC: Immunohistochemistry
MMPs: Matrix metalloproteinases
MITF: Microphthalmia-associated transcription factor
SEM: Standard error of the mean
