# Supplementary material for: Arcyriaflavin A, a cyclin D1/CDK4 inhibitor, suppresses tumor growth, migration, and invasion of metastatic melanoma cells
Source: Cancer Cell Int. 2025 Feb 13;25:42. doi: 10.1186/s12935-025-03675-4 (PMC11827473; doi:10.1186/s12935-025-03675-4)
Supplement: Supplementary file 1 — Supplementary Material 1 [file 12935_2025_3675_MOESM1_ESM.pdf]

Supplementary Material

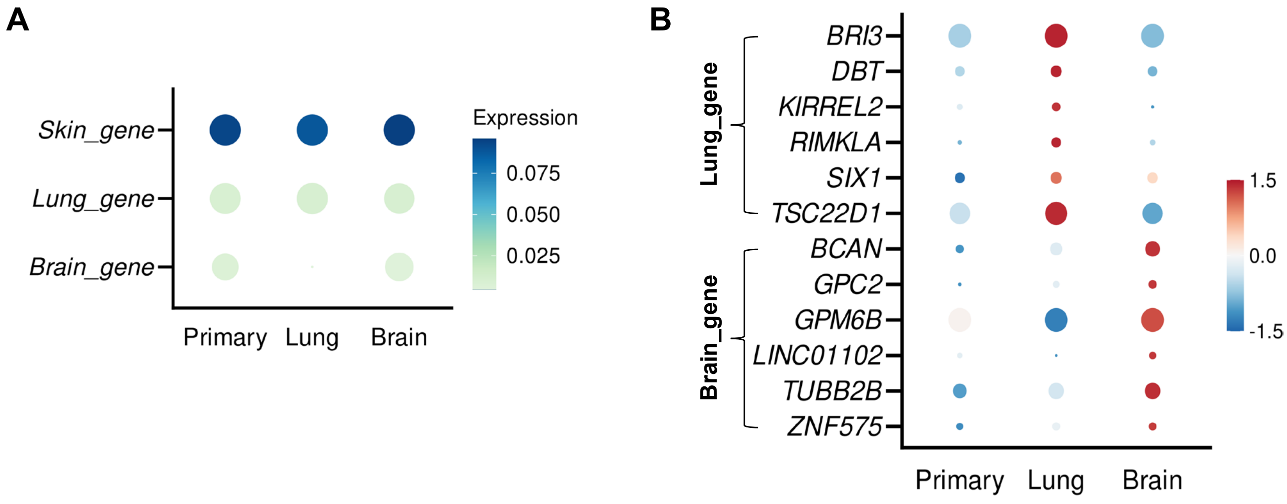

Additional file 1: Figure S1 Tissue-specific gene expression analysis in melanoma cell lines (A) Dot plot showing enrichment scores for the expression levels of tissue-specific genes, including skin (144 genes), lung (125 genes), and brain (233 genes), in the primary melanoma cell line (WM239A) and its metastatic variants derived from lung (113/6-4L) and brain (131/4-5B1 and 131/4-5B2). (B) Dot plot illustrating the average expression levels of representative lung- and brain-specific genes in the same cell lines.

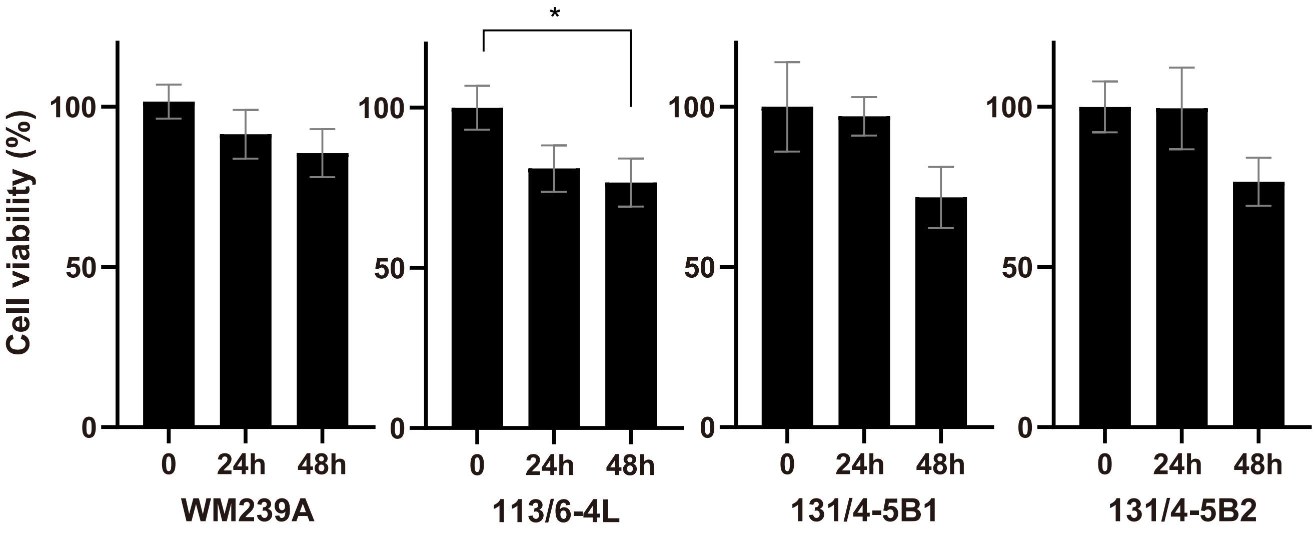

Additional file 1: Figure S2 Cell viability following 10 µM ArcA treatment at 24 h and 48 h. Four melanoma cell lines were seeded at a density of 8×10^3 cells per well in 96 well plates. WST assays were performed at each time point to assess cell viability. DMSO was used as the control. The percentages of viable cells are presented relative to the control group.

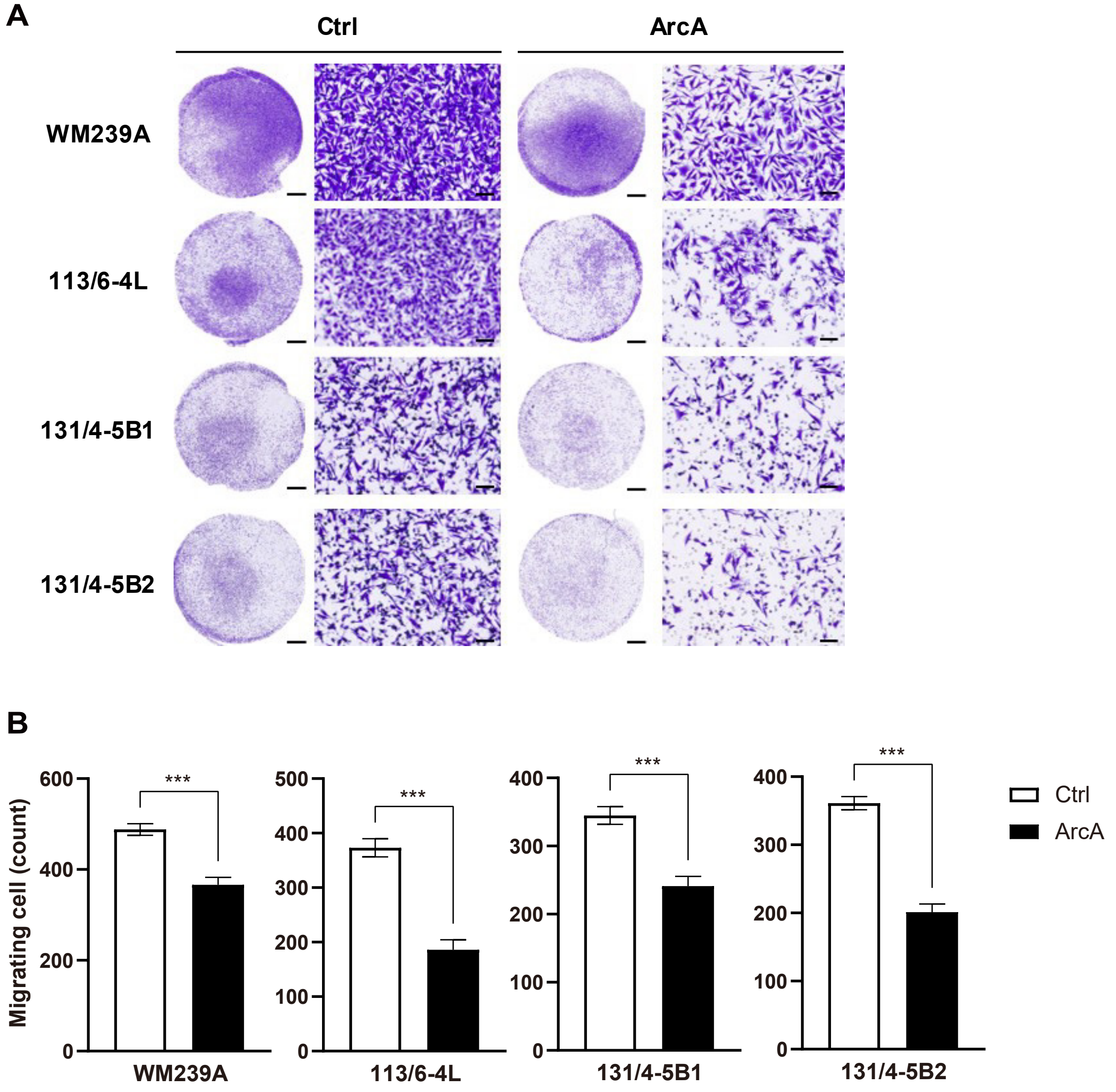

Additional file 1: Figure S3 Inhibition of migratory properties by ArcA as assessed by the transwell migration assay. Transwell migration assay showed a significant reduction in the number of migrating cells following a 48 h treatment of ArcA treatment. Lower-magnification images (1.5×) are shown in the left panels, while higher-magnification images (20×) are displayed in the right panels. Scale bars: 1000 μm (left panels) and 50 μm (right panels).

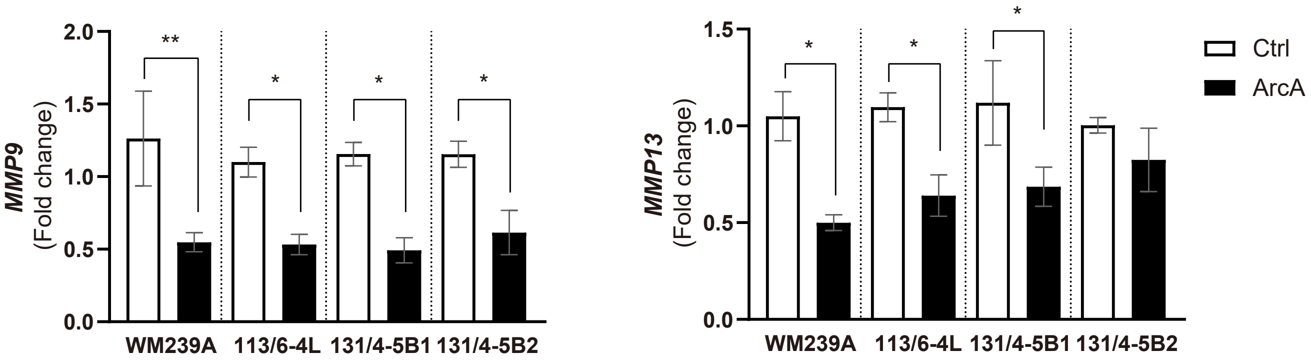

Additional file 1: Figure S4 Reduction of MMP9 and MMP13 mRNA expression levels following ArcA treatment. qRT-PCR analysis was performed to evaluate MMP9 and MMP13 mRNA expression levels in melanoma cells treated with 10 µM ArcA or an equivalent volume of DMSO (control). Relative MMP expression level were calculated using the $2^{-\Delta\Delta CT}$ method. Data are expressed as the mean ± SEM, with statistical significance determined by a *t*-test (*$p < 0.05$, ** $p < 0.01$). All experiments were validated through three independent repetitions.

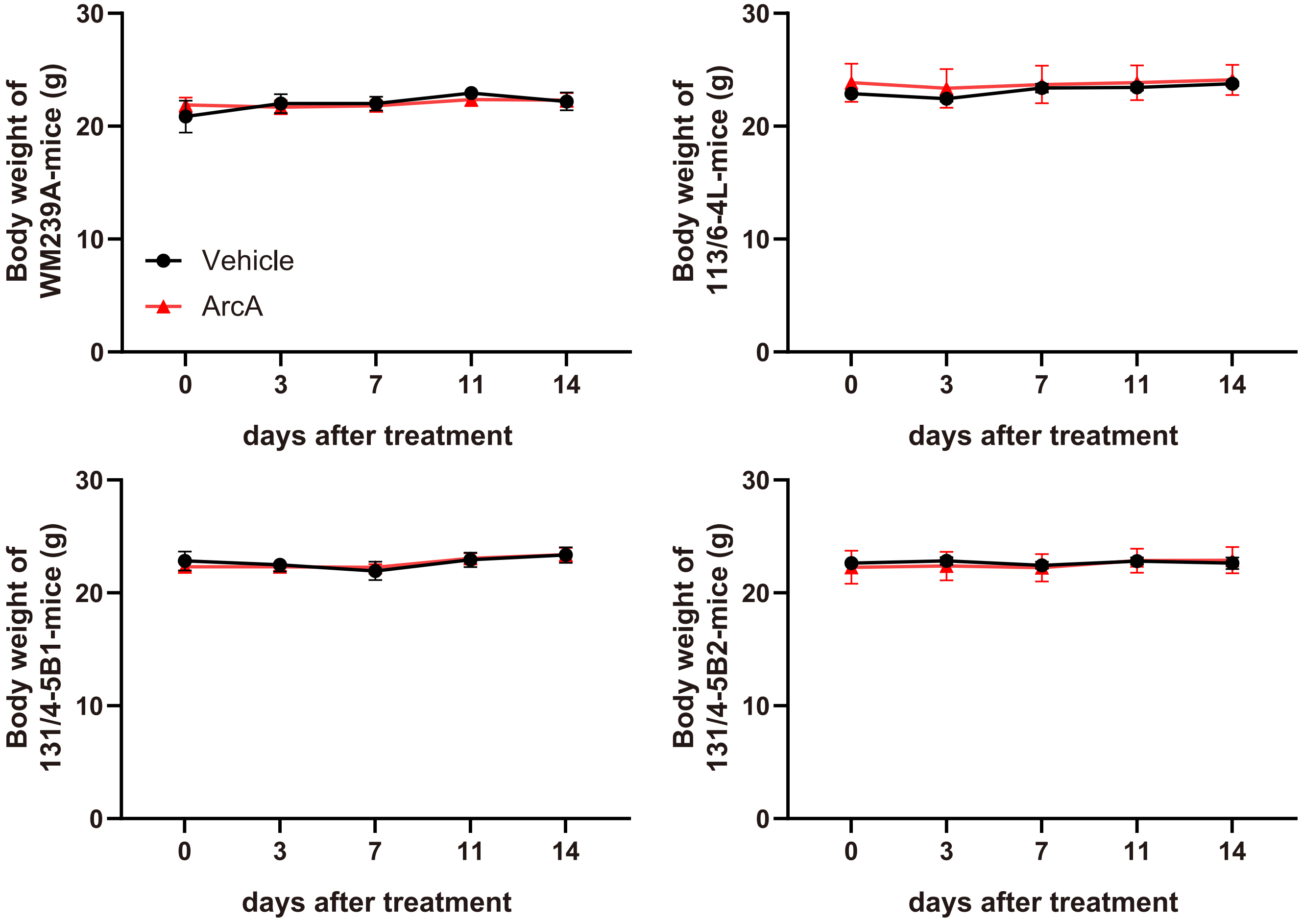

Additional file 1: Figure S5 Effect of ArcA on body weight in a mouse xenograft model. Body weight measurements were taken biweekly for all mice. Data are presented as mean values ± SEM, and statistical analysis was performed using multiple *t*-test. No significant differences in body weight were observed between the treatment and control groups.
